# A 125 million-year-old freshwater isopod shines new light on the origin of subterranean freshwater species

**DOI:** 10.1098/rsos.241512

**Published:** 2025-04-02

**Authors:** Mario Schädel, Dany Azar, Layla El Hajj, Sibelle Maksoud, Ninon Robin

**Affiliations:** ^1^ Senckenberg Centre for Human Evolution and Palaeoenvironment, University of Tübingen, Rümelinstr. 23, Tübingen 72072, Germany; ^2^ State Key Laboratory of Palaeobiology and Stratigraphy, Nanjing Institute of Geology and Palaeontology, Chinese Academy of Sciences, Nanjing, Jiangsu 210008, People’s Republic of China; ^3^ Department of Natural Sciences, Faculty of Sciences II, Lebanese University, Fanar, PO Box 26110217, El-Matn, Lebanon; ^4^ Institut des Sciences de la Terre, Sorbonne Université, ISTeP, UMR 7193, CNRS, 4 Place Jussieu, Paris 75005, France; ^5^ Centre de Recherche en Paléontologie-Paris, CR2P, UMR 7207, CNRS, Sorbonne Université, 4 Place Jussieu, Paris 75005, France; ^6^ Royal Belgian Institute of Natural Sciences, 31 Vautierstreet, Brussels 1000, Belgium; ^7^ Géosciences Rennes (UMR 6118), Université de Rennes, CNRS, 263 Général Leclerc Avenue, Rennes 35042, France

**Keywords:** cretaceous, Cirolanidae, dysodile, multispectral imaging, palaeoenvironmental reconstruction, salinity

## Abstract

Here, we report fossil isopods preserved in laminated oil-shale mudstone (dysodile) from the Lower Cretaceous of Lebanon (Lower Barremian, 125 Ma, Grès du Liban Alloformation, Jezzine District). Based on a variety of proxies, their palaeoenvironments are determined to have been a shallow freshwater lake. The fossil isopods were studied using modern imaging techniques, such as multispectral imaging and photometric stereo, allowing for a detailed comparison of these specimens with comparable extant and fossil taxa. The conspecific fossils are herein recognized as remains of a new species*—*†*Dysopodus gezei* gen. et sp. nov.—of uncertain affinity within Cymothoida and bearing a strong resemblance to its non-parasitic lineages (Cirolanidae). A conspicuous pleotelson and uropod morphology set it apart from most species, with the notable exception of †*Pseudoplakolana chiapaneca* gen. nov. et comb. nov. from the Cretaceous of Mexico, originally attributed to an Australasian lineage (herein disputed). So far, the biogeographical distribution of the peri-Mediterranean underground fauna has predominantly been explained through a passive isolation process of former marine species, driven by regressing coastlines. Stemming from a freshwater lake environment, the 125 million-year-old fossils from Lebanon provide an unconventional perspective on the evolutionary origin of extant cave- and groundwater-dwelling cymothoidans.

## 1. Introduction

### 1.1. Natural history and ecology of Cymothoida

Isopoda is a diverse group of malacostracan crustaceans that comprises more than 10 000 described extant species [1]. Most extant species are found in various marine environments ranging from the deep sea to sandy beaches and rocky shores. Isopoda can be considered a primarily marine group, with the most recent common ancestor of all isopods most likely being marine [2]. However, there are also many isopod species that live outside of the marine realm. A species-rich ingroup of Isopoda—Oniscidea—houses more than 3800 species, of which most live in various fully terrestrial habitats [1,3]. Apart from marine and terrestrial environments, isopods also inhabit brackish and freshwater environments, with around 1000 described species living in freshwater [4]. Isopods have colonized freshwater habitats multiple independent times, resulting in a variety of distinct taxa being present in freshwater habitats, ranging from old and, in some cases, very species-rich groups such as Phreatoicidea and Asellidae to single phylogenetically isolated species [4].

Cymothoida is a group of isopods that includes scavengers, predators, micropredators and parasites. Among those, micropredatory and parasitic species likely form a natural group [5,6]. Within Cymothoida, many freshwater species are parasites that likely entered freshwater habitats along with their hosts, which are either fishes or crustaceans [4]. Among representatives of Cymothoida (cymothoidans—not to be confused with cymothoids), there are many that are neither micro-predators nor parasites throughout their life. These are often referred to as ‘Cirolanidae’, a group of morphologically distinct isopods. However, their morphological distinctiveness is based on plesiomorphic character states, and ‘Cirolanidae’ is likely to be paraphyletic with respect to the micropredatory and parasitic forms of Cymothoida [7], but see [5] for potential autapomorphies of ‘Cirolanidae’. While most species of ‘Cirolanidae’ are marine, about 15% can be found in freshwater [8], and about the same proportion lives underground (hypogean, as opposed to epigean) in caves and groundwater [9], with many of the freshwater species living underground. Cave and groundwater species are mostly found in areas that were previously covered with seawater, leading to the interpretation of hypogean species as ‘marine relics’, that is, as species that stayed in place geographically as the seawater retreated [10]. Some authors even saw a correlation between the time since the seawater had retreated and the amount of derived morphological features in the below-ground fauna, suggesting that life in caves and groundwater preserved plesiomorphic features [11,12]. While such a biogeographical model fits many species, there are some species whose geographical occurrence cannot be explained by the former presence of a coastline in the area [9,13].

Apart from amber fossils of terrestrial isopods, the majority of isopod fossils stem from marine palaeoenvironments. Nevertheless, there are also fossils believed to come from isopods that lived in fresh or brackish water [14,15]. Of these, only a few have been attributed to ‘Cirolanidae’. From the Lower Cretaceous Lakota Formation near Piedmont (South Dakota), which is interpreted to have preserved fluvial and lacustrine environments [16], there is †*Cirolana enigma* Wieder and Feldmann, 1992, which is known from a large number of individuals [17]. †*Cymothoidana websteri* Jarzembowski, Wang, Fang and Zhang, 2014 is known from the Lower Cretaceous Lower Weald Clay Formation in Surrey and West Sussex (UK), which is also interpreted to be non-marine [18]. The depositional environment of a recently described species —†*Brunnaega labuttensis* Wilson & Morel, 2022—from the Upper Cretaceous of Le Mans (France) has been interpreted as potentially estuarine [19,20]. For two recently reported amber fossils from the mid-Cretaceous of northern Myanmar (Kachin amber), recognized as two different ontogenetic stages of the same species—†*Electrolana madelineae* Schädel, Hyžný and Haug, 2021 (see [21] for a competing interpretation)—the palaeoenvironmental context does not allow us to infer the salinity of the palaeohabitat [22]. Similarly, no reliable palaeoenvironmental reconstruction can be made for a not formally described fossil from Baltic amber [23].

Herein, we describe two well-preserved isopod specimens from the Lower Cretaceous of Lebanon, with a geological context convincingly suggesting that they lived in freshwater, and discuss their occurrence in the context of the origin of the freshwater isopod fauna in the Mediterranean region.

### 1.2. Geological and palaeoenvironmental setting

**Figure 1.**
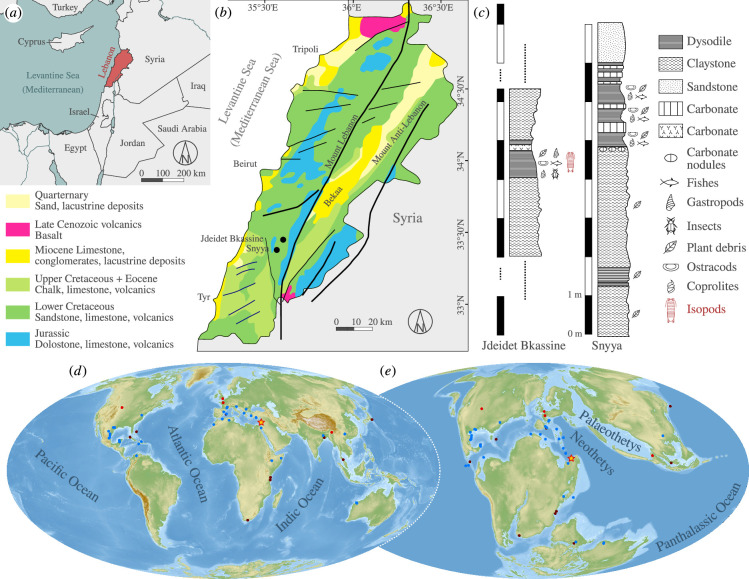
Geographical, geological and palaeogeographic setting. (*a*) Geographical map, showing the location of Lebanon in the Eastern Mediterranean Region. (*b*) Geological map of Lebanon showing the location of Jdeidet Bkassine (origin of the isopod specimen) and the close-by site Snyya, modified after [24]. (*c*) Stratigraphic profile of the field sites Jdeitet Bkassine and Snyya. (*d,e*) Global maps with locations of extant freshwater (blue points) and extant brackish (brown points), fossil presumed non-marine and estuarine isopods (red points) and the herein presented fossil (red-yellow star), Mollweide projection. (*d*) Present-day map. (*e*) Map of the Lower Barremian (125 Ma).

The specimens were found in the Lebanese dysodiles of Jdeidet Bkassine (Jezzine District, South Lebanon Governorate). These layers correspond to finely laminated organic-rich sediments cropping out at five mining localities within the Grès du Liban Alloformation: one in the north of Lebanon, one in the centre and three localities in the south of Lebanon, among which is the Jdeidet Bkassine outcrop (N33°33′0″ E35°34′0″; 713 m elevation). Discontinuities over the Jdeidet Bkassine succession prevent its direct proper logging, though 1-m of the typical dysodiles markedly outcrops sandwiched within 30 m of altered volcanic claystone. The stratigraphic similarities observed in the very close-by locality Snyya (N33°31′5″ E35°33′0″; 875 m elevation), which is to the south of Jdeidet Bkassine (figure 1*b*), allow us to infer a sedimentological continuity between the deposits of both localities. This is based on corresponding dysodile thicknesses among equivalent volcanic clay stones in both sites. The geological profile of the nearby Snyya locality therefore illustrates the facies succession profile in the Jdeidet Bkassine sediments that yielded the specimen. The Grès du Liban Alloformation dates back to the Lower Barremian [25,26] and comprises a series of marine sediments covered by lacustrine carbonates that are rich in charophytes (*Ascidiella reticulata*, *Atopochara trivolvis* var. *triquetra*, *Clavator ampullaceus*, *Clavator delteus*, *Sphaerochara asema* and *Charaxis martinclosasi*) and laterally change to fluvio-deltaic sandstone [27]. This sandstone is believed to be a product of the erosion of pre-existing Carboniferous and Permian sediments, transported by a large river that crossed the Arabian platform and deposited sand in many Arab countries [24]. In addition to sandstones, the Grès du Liban Alloformation contains volcanic rocks, claystone, lignite, amber and dysodiles. Recent studies aimed to understand the origin and the depositional environment of these dysodiles [28–30]. All evidence points to a series of small, shallow lakes and/or swamps located in proximity to volcanic edifices. Moreover, to explain the presence of these lakes in the sandstone unit, two hypotheses were suggested. They could be the result of abandoned river meanders, or—because volcanism appears to have contributed to events of deposition nearby—of episodes of lava blocking the flow of previously existing rivers, resulting in the formation of lakes.

The depositional environment of the dysodile layers in Jdeidet Bkassine can be reconstructed using abiotic and biotic characteristics of the sediment. Geochemical analyses, such as Rock–Eval pyrolysis and elemental analysis, indicate that the organic matter composing the dysodiles is derived from algae and bacteria that were deposited in a lacustrine environment. The total organic carbon and sulfur contents indicate a freshwater lake as the origin of the sediment [28,31]. Microscopic profiles of this organic matter (including petrography) show the dominance of highly fluorescent amorphous organic matter and lamalginites [28], which supports a lacustrine algal origin [32].

The dysodiles yielded no salinity biomarkers but biomarkers indicative of a strong freshwater algal and bacterial influence in the deposition of organic matter. The algal influence is attested by the dominance of short-chained *n*-alkanes in the range of 15–19 carbon atoms [33,34] and the bacterial influence by long-chained *n*-alkanes in the range of 25–31 carbon atoms, respectively [34]. An indicator for a lacustrine environment was the ratio between $C_{31}$R and $C_{30}$ hopane [34], which was greater than 0.25. Pristane/$nC_{17}$ values >0.6 indicate terrigenous rather than marine conditions, the Gammacerane index and the 17α(H), 21α(H)-homohopane co-elution [34], and the regular $C_{27}$–$C_{29}$ steranes ternary diagram; and the indicators of freshwater are the contributions of some non-marine algae to the $C_{27}$ and $C_{31}$ *n*-alkanes, causing the increase of the terrigenous to aquatic ratio [35,36].

An overall study of the faunal assemblage also points towards a freshwater depositional environment. Pulmonate snails, such as *Lymnaea* sp., suggest the presence of freshwater or nearby wetlands. The presence of fossil mayfly larvae (Ephemeroptera) in the sediment suggests a freshwater environment due to the low salinity tolerance in extant species [30]. Fossils stemming from species in two groups of ostracods, *Cypridea* and *Zonocypris,* are also indicative of the palaeoenvironment ([29], p. 202). All species in *Cypridea* are now extinct, but its representatives are generally recognized to have lived in freshwater, but also in athalassic saline temporary and permanent water bodies [37,38]. The extant group *Zonocypris* is indicative of freshwater and waterbodies with low alkalinity and low dissolved inorganic carbon content [39].

The available geological, palaeontological and petrochemical evidence consistently indicates that the dysodile, which yielded the herein-studied fossil, was deposited in a small freshwater lake in the vicinity of volcanic edifices. The absence of salinity biomarkers and fossils of marine organisms, as well as the presence of fossils of organisms for which a low salinity tolerance is presumed, excludes a direct marine influence in the immediate environment, although a nearby coastline is suggested for the depositional environment by the geological and palaeogeographic setting (figure 1*d*).

## 2. Material and methods

### 2.1. Material

The two fossils presented herein were excavated in the years 2003 (CRU 63124/1) and 2023 (CRU 63124/2). Both fossils are located on thin layers of finely laminated mudstone. One specimen (CRU 63124/1) was mounted onto a 75 mm × 26 mm glass microscope slide to enhance its mechanical stability. Both specimens are housed at the Natural History Museum of the Faculty of Sciences II at the Lebanese University in Fanar.

### 2.2. Microscopy

High-resolution micrographs of the fossils were created with the aid of a Keyence VHX 7000 digital microscope with inbuilt focus merging and panoramic stitching functionalities. Coaxial white light in a cross-polarizing filter setup was used to reduce reflections and increase the contrast between the fossil and the sediment matrix. Additionally, ring and partial ring light illumination (white light) were for recording additional images, including such that distinct shadows (not figured but used for the drawing). In some cases, two or three images of the same field of view with different exposure settings were recorded and merged using enfuse (https://enblend.sourceforge.net, GPL v. 2 license) to avoid underexposure and overexposure by enlarging the dynamic range of the images.

### 2.3. Multi-light imaging

One fossil (CRU 63124/1) was photographed with different illumination settings (different main illumination directions) in order to produce a surface shape model (‘photometric stereo’ [40]). For this, the fossil was sequentially illuminated using a custom-built light dome setup [see [41] for detailed specifications]. The images were captured with a Nikon D7200 DSLR camera in combination with a Laowa 100 mm f/2.8, infinity-focus−2× macro lens. Multiple illumination sequences were recorded for different relative heights of the focal plane (focus-stacking). The tip of a ballpoint pen was used as a reference object for approximating the light directions using specular highlights on the spherical object. This was done using the graphical user interface of Relight (https://github.com/cnr-isti-vclab/relight, GPL v. 3 license [42]), producing a text file with coordinates for the illumination directions. The image processing was automated with a bash script, facilitating the program relight-cli [42] for creating normal map representations of the surface shape of the fossil, align_image_stack (https://hugin.sourceforge.io, GPL v. 2 license) for aligning the focus stack of normal maps, and enfuse (https://enblend.sourceforge.net, GPL v. 2 license) for merging the focus stack (extended depth of field). For a detailed discussion of this technique, see [41].

#### 2.3.1. Luminescence and multispectral imaging

The multispectral microimaging of one of the specimens (CRU 63124/1) was performed at the research facility IPANEMA (SOLEIL Synchrotron, Orsay, France). The concept of multispectral imaging is based on the collection of reflection and luminescence images from various spectral ranges using an innovative imaging setup (see [43]). The setup consists of a low-noise 2.58-megapixel back-illuminated monochrome sCMOS camera (PRIME 95B 25 mm, Teledyne Photometrics) with a high sensitivity from 200 to 1000 nm wavelength. The camera was fitted with a UV–VIS–IR 60 mm 1:4 Apo Macro lens (CoastalOptics), in front of which a filter wheel was positioned. The filter wheel held eight interference band-pass filters (Semrock) to collect images in eight spectral ranges from 435 to 935 nm. The illumination was provided by 16 LED lights with emission spectra ranging from 365 up to 700 nm wavelength (CoolLED pE-4000). These were coupled to a fibre-optic ring light with the aid of a liquid light guide. From the 96 possible combinations of LED illumination spectra and band-pass filters in front of the lens, three combinations were selected to compose a false-colour RGB image (see respective figure caption). For each illumination–detection combination, multiple images with differing relative heights of the focal plane (focus-stacking) and differing fields of view (panoramic imaging) were recorded. In-focus panoramic images were created from these using ImageJ (https://imagej.net/ij/, public domain [44]) and Microsoft Image Composite Editor (proprietary, usage free of charge). The three resulting greyscale images were then combined into a single false-colour RGB image using GIMP (https://gimp.org, GPL v. 3.0 license).

### 2.4. Collection of habitat data for extant species

A list of extant freshwater and brackish water isopod species was retrieved from the WoRMS online database [8], available at https://www.marinespecies.org/, by downloading a ‘taxon list’ via the advanced taxa search dialogue that includes columns for the main environment types. Geographical occurrences of species from the taxon list and of some additional species, including extinct species, were retrieved from the literature, largely by inspecting publications linked in the WoRMS online database (electronic supplementary material, files S1 and S2). Coordinates were recorded in QGIS (https://qgis.org, GPL v. 2 license) and exported as an ESRI shapefile. The palaeogeographic and present-day global maps were created in GPlates (https://www.gplates.org, GPL v. 2 license) using a digital palaeo-elevation model [[45], CC-BY 4.0 license]. The palaeo-coordinates were reconstructed in GPlates by assigning the present-day coordinates to tectonic plate polygons and applying a rotation model [[46], CC-BY 4.0 license].

### 2.5. Image processing and graphic design

The bitmap images were optimized for colour, contrast and sharpness using GIMP. Inkscape (https://inkscape.org, GPL v. 3.0 license) was used to produce the line drawings and to assemble the figure plates. The circular colour legends accompanying the normal maps simulate the normal map appearance of a (half-) spherical object as if captured and processed like the corresponding image [47,48].

## 3. Results

### 3.1. Systematic position

Isopoda Latreille, 1817

Scutocoxifera Dreyer and Wägele, 2002 [49]

Cymothoida Wägele, 1989 [7]

### 3.2. †*Dysopodus* gen. nov.

urn:lsid:zoobank.org:act:01DBEA7D− 7022− 4904− 8FE0− 677E8C4E68F3

**Type species**. †*Dysopodus gezei* gen. et sp. nov.

**Etymology**. The name refers to the lithology of the sediment matrix—dysodile—in which the type specimens of the type species are preserved. The second part of the name refers to the word Isopoda. The Latin gender is masculine.

**Diagnosis**. As for the type species (monotypic).

### 3.3. †*Dysopodus gezei* sp. nov.

urn:lsid:zoobank.org:act:DD5C57B3− 4F7D− 4EBE-A76C-E7569ECA8E42

**Holotype**. CRU 63124/1

**Paratype**. CRU 63124/2

**Etymology**. The specific epithet honours the accomplishments of Raymond Gèze, who explored the dysodile layers at the field site over many years.

**Type locality and stratum**. Jdeidet Bkassine, Jezzine District, South Governorate, Lebanon (N33◦33′0″ E35◦34′0″; 713 m elevation); Grès du Liban Alloformation; Lower Barremian (Lower Cretaceous).

**Figure 2.**
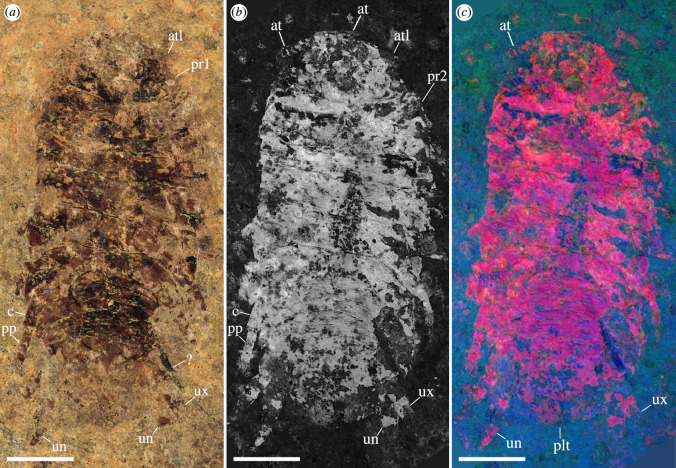
†*Dysopodus gezei* gen. et sp. nov., holotype CRU 63124/1, micro-photographic images. (*a*) coaxial white light, cross-polarized. (*b*) visible light-induced luminescence, excitation maximum 435 nm (violet), collected light 835 nm ± 70 nm (infrared). (*c*) false-colour image derived from multispectral imaging, blue channel: excitation maximum 385 nm (UV), collected light 360 nm ± 23 nm (UV), green channel: excitation maximum 490 nm (cyan), collected light 571 nm ± 72 nm (yellow), red channel: excitation maximum 435 nm (violet), collected light 835 nm ± 70 nm. Scale bar: 3 mm. at, antenna; atl, antennula; c, carpus; plt, pleotelson; pp, propodus; pr1−2, pereonites 1−2; un, uropodal endopod; ux, uropodal exopod; ?, unknown structure, likely not part of the isopod.

**Figure 3.**
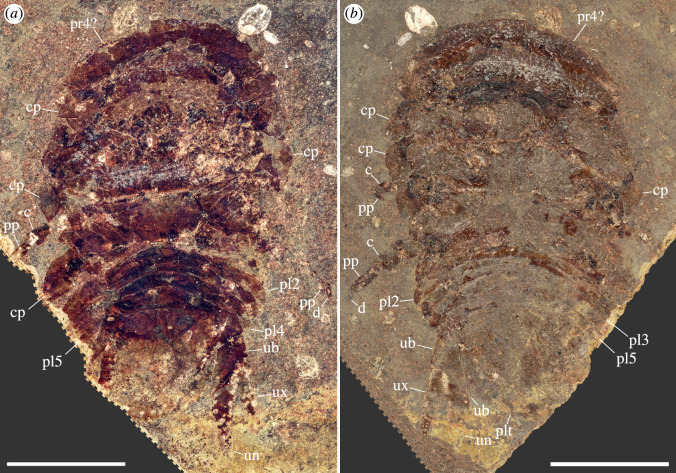
†*Dysopodus gezei* gen. et sp. nov., paratype CRU 63124/2, part and counterpart, micro-photographic images, dashed margins denote image masking for aesthetic reasons. (*a*) Part, coaxial white light, cross-polarized. (*b*) Counterpart, white light, ring light illumination. Scale bars: 5 mm. c, carpus; d, dactylus; cp, coxal plate; pl2−5, pleopods 2−5; plt, pleotelson; pp, propodus; pr4?, possible pereonite 4; ub, uropodal basipod; un, uropodal endopod; ux, uropodal exopod.

**Figure 4.**
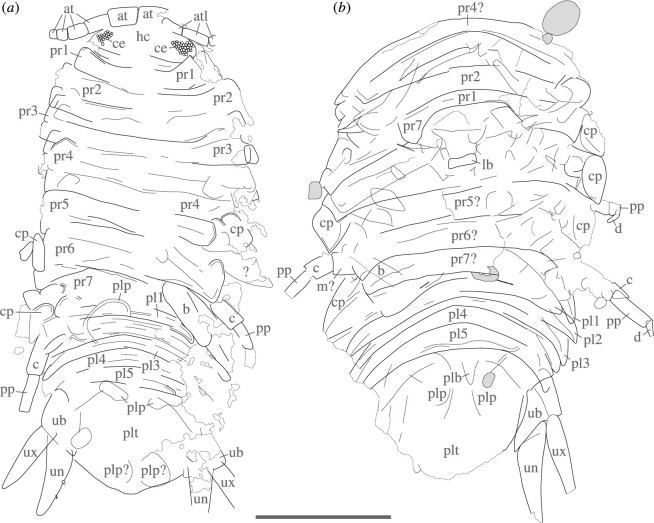
†*Dysopodus gezei* gen. et sp. nov., interpretative line drawings based on multiple image modalities. (*a*) Holotype CRU 63124/1. (*b*) Paratype CRU 63124/2. Scale bar: 5 mm. at, antenna; atl, antennula; b, basis; c, carpus; ce, compound eyes; cp, coxal plate; d, dactylus; lb, labrum; m?, possible merus; pl1−5, pleopods 1−5; plp, pleopod; plt, pleotelson; pp, propodus; pr1−7, pereonites 1−7; ub, uropodal basipod; un, uropodal endopod; ux, uropodal exopod; ?, unknown/uncertain structure.

**Description**. Body elongate, slightly more than twice as long as wide, oval overall shape in dorsoventral projection, widest at approximately half of length (figures 2 and 3). Total length of holotype is 17.9 mm, total width of holotype is 8.0 mm; paratype is considerably larger, likely up to 25 mm in total length (figure 4*a* versus figure 4*b*; but see discussion on taphonomy).

**Figure 5.**
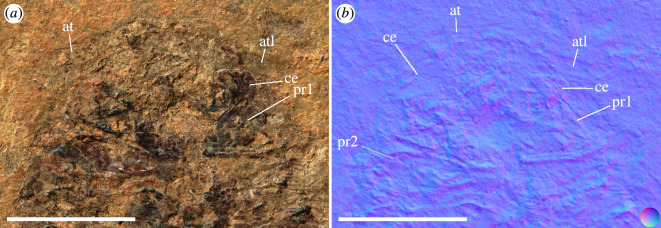
†*Dysopodus gezei* gen. et sp. nov., holotype CRU 63124/1, detail of the head region. (*a*) Micro-photographic image, white light, ring light illumination. (*b*) Normal map derived from photometric stereo, multiple focus planes merged; for orientation, see reference sphere. Scale bar: 3 mm. at, antenna; atl, antennula; ce, compound eye; pr1−2, pereonites 1−2.

Head distinctly wider than long, roughly semicircular in dorsal view (neglecting parts covered by subsequent tergites). Eyes located on lateral sides of head, left and right eye well separated, from lateral margin of head extending medially to approximately halfway to mid-line of head, each eye consisting of at least 35−40 ommatidia, ommatidia arranged in rows parallel to margin of head (figure 5). Antennula with three elements preserved. Most proximal preserved antennula elements corresponding to penultimate peduncle element, elongate with straight abaxial sides; ultimate peduncle element is slightly longer than the preceding element and approximately same width. The first flagellum element (the distal-most preserved antennula element) is approximately the same width as the preceding peduncle elements but much shorter, approximately as wide as long. Antenna with distal-most three peduncle elements and first two flagellum elements preserved. Proximal-most preserved antenna element corresponding to third peduncle element (e.g. [50]). Third peduncle element is wide, approximately twice as wide as the peduncle elements of the antennula, and slightly less than twice as long as wide. The fourth peduncle element is slightly shorter and distinctly narrower than the preceding element. The fifth (ultimate) peduncle element is approximately as wide but distinctly shorter than the preceding element. The first flagellum element is slightly narrower than preceding peduncle element, approximately as wide as long. The second flagellum element is similar in shape to the preceding element but slightly shorter and narrower. Labrum is short and wide, approximately three times as wide as long, with straight proximal and distal margins.

The trunk is composed of two distinguishable tagmata: the pereon (post-ocular segments 7−13) and the pleon (post-ocular segments 14−19), the last pleon segment conjoined with the telson, forming the pleotelson. Pereonite 1 wider than head, anterior margin distinctly concave, anterolateral aspects encompassing posterolateral parts of head, lateral margins approximately straight, posterior margin slightly convex. Pereonites 2−7 with coxal plates. Pereonite 2 is similar in shape to pereonite 1 but considerably wider and longer and with a much less concave anterior margin. Pereonites 3 and 4 are similar to pereonite 2; pereonite 3 is slightly wider than pereonite 2. Coxal plate of pereon segment 3 has a distinctly angled posterodistal corner. Coxal plates of pereon segments 4 and 5 with rounded anterodistal margins and distinctly angled posterodistal corners with straight lateral margins. Pereonites 5 and 6 are similar in dimension to the preceding tergites, with anterior and posterior margins about straight. Pereonite 7 is approximately as long and as wide as preceding tergites (lateral margins not well preserved). Pereopods not unequivocally attributable to trunk segments. Pereopods of mid-trunk region, each with straight to mildly curved propodus. More posterior pereopods with basis wide but elongate, slightly expanding in width towards the distal end, ischia elongate, more than twice as long as wide, merus similar in width but much shorter (approximately one-third longer than wide), lateral aspect slightly longer than median aspect, possibly forming a faint laterodistal protrusion, carpus much slenderer than merus (approx. 1.5 times as long as wide), propodus elongate and about straight, tapering towards the distal end, distal end of dactylus and claws not apparent.

Pleon is much shorter than pereon, approximately 0.35 times the length of pereon. Pleon tergites are similar in shape and size, much shorter than pereonite 7, anterior margins convex, posterior margins concave, anterior–posterior aspect decreasing laterally, with widely rounded anterolateral aspects and with distinct, narrowly rounded to angled posterolateral tips. Pleopod morphology is not conclusively apparent from holotype and paratype, pleopod basipod possibly with mediodistal protrusion (paratype).

Pleotelson roughly triangular in shape, posterior margin entire (without apparent dents or spines), posterior margin with approximately straight posterolateral portions and with rounded posteromedial margin; exact dimensions not clearly observable, considerably to slightly wider than long, holotype lacking anterior aspects of lateral margins, paratype without posterior margin and with only one intact lateral aspect.

Uropod distinctly extends beyond the level of the posterior tip of the pleotelson. Uropodal basipod sub-triangular, wider than long, distinctly wider in the distal part, lateral margin approximately straight, medial margin straight to slightly convex, lateral and medial margin diverging distally at about a 20°−30° angle, distal margin straight to slightly convex, posteromedial corner distinctly angled. Uropodal exopod elongate, lanceolate, more than three times longer than wide, lateral margin slightly convex, medial margin with a slightly concave section, distal tip angled. Uropodal endopod slightly longer than exopod, lanceolate, lateral margin slightly but conspicuously concave, medial margin rounded and distinctly convex with at least two (but presumably more) robust spines.

**Diagnosis**. The body approximately twice as long as wide, the eyes well developed and located on the dorsolateral sides of the head, the antenna with five peduncle elements and wide third peduncle element, pereonite 1 with distinctly concave anterior margin (lateral aspects partly encompassing head), pleotelson at least slightly wider than long and roughly triangular in shape, pleotelson posterior margin with approximately straight posterolateral portions and with rounded median portion, uropod extending well beyond posterior margin of pleotelson, uropodal endopod and exopod lanceolate, uropodal endopod slightly longer than exopod and with slightly but conspicuously concave lateral margin.

**Remarks**. Note that it is not clear from the fossils whether the visible ommatidia are located on the dorsal or ventral side of the head.

### 3.4. †*Pseudoplakolana* gen. nov.

urn:lsid:zoobank.org:act:3EA535DC− 9FBC− 4DB2− 82E1-AE543BB4F03C

**Type species**.†*Plakolana chiapaneca* Bruce, Serrano-Sánchez, Carbot-Chanona & Vega, 2021.

**Etymology**. The name refers to the superficial similarity to the species of *Plakolana* Bruce, 1993 emphasized in the original description of †*Plakolana chiapaneca*.

**Diagnosis**. As for the type species (monotypic).

### 3.5. †*Pseudoplakolana chiapaneca* (Bruce, Serrano-Sánchez, Carbot-Chanona & Vega, 2021) comb. nov.

**Type material**. IHNFG−5934 (holotype), IHNFG−5935–IHNFG−5944 (paratypes).

**Type locality and stratum**. El Espinal quarries, Chiapas, Mexico; Sierra Madre Formation; Aptian (Lower Cretaceous).

**Remark**. The species has originally been attributed to *Plakolana* by excluding several possible affinities, ultimately leading to a ‘best fit’ attribution based on a combination of characters that also occur in species not attributed to *Plakolana* [51]. Furthermore, the extant species do not share the conspicuous uropod morphology of the Mexican fossil. The original interpretation would place †*Plakolana chiapaneca* as the only extinct species among extant species from Australia, Tasmania and Papua New Guinea [51]. Because this would imply far-reaching consequences for the biogeographical origin and the geological age of *Plakolana*, the species is herein assigned a new generic name.

## 4. Discussion

### 4.1. Fossil preservation and conspecificity

**Figure 6.**
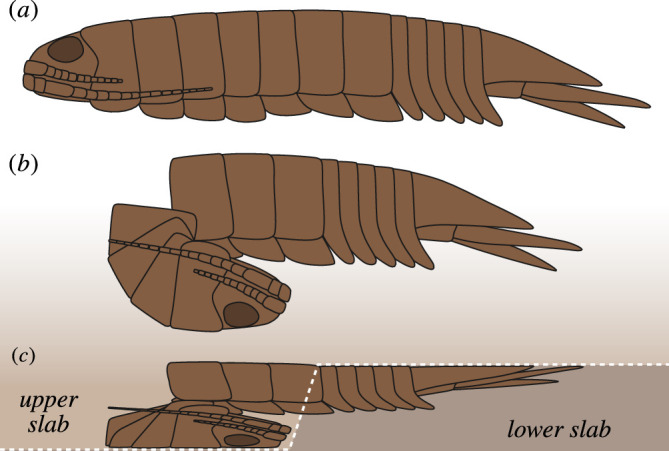
Taphonomic model assumed for the preservation of the paratype CRU 63124/2. (*a*) Isopod specimen in living condition, lateral view. (*b*) Specimen at the time of sediment deposition. (*c*) Specimen post-burial showing the effect of sediment compaction (compaction much stronger in the actual fossil). White dashed line depicting the cleavage surface between the two dysodile slabs (drastically simplified).

The two herein-described fossil specimens are highly compressed, bearing only a very shallow relief. The holotype is preserved such that most remains are on one dysodile slab, with the exposed surface providing a ventral view of the fossil. Nevertheless, because of the strong compression, also dorsal features are visible, especially under grazing light conditions. Therefore, the exact location of the eyes is not apparent, as the preserved ommatidia could stem from either the dorsal or ventral side. While, in most extant isopod species, the eyes are visible in dorsal view, there are numerous exceptions to this, recorded from various lineages, especially within ‘Cirolanidae’ (e.g. [50]). In contrast, the sclerite remains of the paratype are complementary with respect to the part and counterpart slab. Additionally, the preservation of the paratype is complicated by the posture and orientation of the specimen at the time of deposition (figure 6). Before the sediment experienced compression, the specimen appears to have been dorsoventrally bent (figure 6*b*). Note that this does not necessarily reflect the original range of motion, as the deposition might have happened well after death.

Interestingly, the preservation of the paratype is best explained by a separation of pereon tergites 4 and 5, where in extant isopods the anterior and posterior exuvia parts separate. However, most isopods exclusively moult in a biphasic manner, with the posterior exuvial part being shed first ([52–54; but see exceptions in [55–57]). Therefore, the preservation does not allow for the identification of the paratype as an exuvium without questioning the ubiquity of biphasic moulting in isopods.

The differences in preservation between the two herein presented fossil specimens complicate their detailed comparison, as only a few biologically relevant aspects of their morphology can be directly compared. Nevertheless, the lack of apparent differences, except for the larger overall size of the paratype, together with the distinct shape and relative size of the uropods shared by both specimens, strongly suggests that the two specimens are conspecific.

### 4.2. Systematic affinity of the fossil

The specimen can be easily identified as a representative of Isopoda, based on a number of features. None of them are exclusive to Isopoda; yet, the combination of several features is highly characteristic for Isopoda. These features include a dorsoventrally flattened body (not present in all isopods), the lack of a carapace, a pereon with seven segments bearing walking or grasping appendages, a pleon with five free segments, a pleotelson (conjoined pleon tergite 7 and telson) and uropods (appendages of pleon segment 6) [7,58]. The presence of coxal plates (conjoined structures of tergites and coxae) unambiguously identifies the fossil specimens as representatives of the species-rich isopod ingroup Scutocoxifera [49]. The roughly triangular shape of the uropod basipod, resulting from a large posteromedial process, has been interpreted as apomorphic for Cymothoida Wägele [7]. Other aspects apparent from the fossils, such as the overall body shape and the leg morphology, are also conforming with extant species of Cymothoida.

Cymothoida comprises extant species with a variety of largely carnivorous feeding habits, such as predation, scavenging and different forms of parasitism [5]. Within Cymothoida, parasitism clearly represents a derived feeding mode, with the extant micropredatory and parasitic species of Aegidae, Corallanidae, *Tridentella* and Cymothoidae (micropredators and parasites of fish) likely forming a monophylum [2,5], which might also include the groups Epicaridea (parasites of crustaceans; [7,59]) and Gnathiidae (micropredators of fish; [6]).

The morphology preserved in the fossils is consistent with predatory and scavenging forms of Cymothoida, recognized in the literature as ‘Cirolanidae’. However, ‘Cirolanidae’ lacks strong autapomorphies (but see [5]), especially such that would be visible in a compression fossil. Furthermore, with the accessible morphological features of the two fossils at hand, we could not identify any ingroup of ‘Cirolanidae’, to which †*Dysopodus gezei* gen. et sp. nov. could be attributed on phylogenetic grounds. Therefore, the possible non-‘Cirolanidae’ ingroups of Cymothoida are also addressed here briefly.

Anthuridea can be excluded, as the body of †*Dysopodus gezei* gen. et sp. nov. is not distinctly elongated and vermiform; also, the coxal plates in †*Dysopodus gezei* gen. et sp. nov. are large and protrude from the trunk, unlike in anthurideans [7]. Gnathiidae and †*Urda* are characterized by pereon segment 1 being functionally part of the head (pereonite 1 distinctly shortened or conjoined with the head capsule) [60], which is not the case for †*Dysopodus gezei* gen. et sp. nov., where pereonite 1 laterally encompasses the head. The rather straight and inconspicuous dactyli and the triangular shape of the uropod basipod in †*Dysopodus gezei* gen. et sp. nov. (figure 4*b*) excludes Cymothoidae and Epicaridea as possible affinities, where one would expect a robust hook-shaped complex of dactylus and claw on pereopods 1−7 and a non-triangular uropod basipod, lacking a distinct medial process [6,7]. It is also fairly unlikely that †*Dysopodus gezei* gen. et sp. nov. is a representative of Aegidae, where hook-shaped distal leg elements are restricted to pereopods 1−3 [61] as these leg elements have a high preservation potential [62] and should therefore be visible, at least as impressions, in the herein described fossils.

As stated above, it was not possible to identify †*Dysopodus gezei* gen. et sp. nov. as representative of any monophyletic ingroup of Cymothoida. By this, we also deliberately refrained from attributing †*Dysopodus gezei* gen. et sp. nov. to ‘Cirolanidae’ despite the similarity to its extant attributed species. One reason for this decision is the lack of convincing support for the monophyly of ‘Cirolanidae’ with respect to its extant species [7,59]. The second reason, independent from the first, is that the ancestor(-s) of the micropredatory and parasitic forms of Cymothoida have been reconstructed to be morphologically similar to extant species of ‘Cirolanidae’ (e.g. [59,63]). This renders it virtually impossible to determine whether a fossil stems from an organism that is more closely related to *Cirolana cranchii*—the type species of the type genus of ‘Cirolanidae’—or to any species of Aegidae or Cymothoidae, especially considering the fragmentary nature of the morphological information preserved in fossils.

### 4.3. Comparison with the fossil record

**Figure 7.**
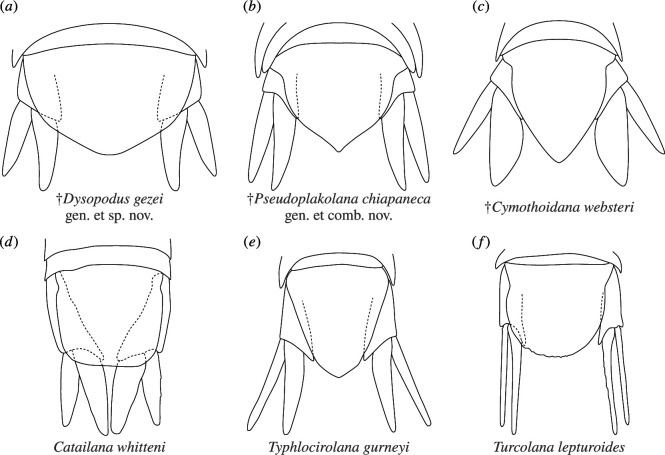
Drawings of the pleotelsonic regions of fossil (upper row) and extant (lower row) isopods deemed especially relevant for comparison; dashed lines denote structures that are overlain by other body parts. (*a*) Reconstructive drawing of †*Dysopodus gezei* gen. et sp. nov. (*b*) Reconstructive drawing of †*Pseudoplakolana chiapaneca* comb. nov., based on [[51], figures 2*a*, *d* and 3*q*]. (*c*) Reconstructive drawing of †*Cymothoidana websteri*, based on [[18], figure 4*c*]. (*d*) Drawing of *Catailana whitteni*, redrawn from [[64] figure 2b]. (*e*) Drawing of *Typhlocirolana gurneyi*, redrawn from [[65], pl. 19, figure 61]. (*f*) Drawing of *Turcolana lepturoides*, redrawn from [[66], figures 3 and 16].

†*Dysopodus gezei* gen. et sp. nov. differs distinctly enough from all currently recognized species to warrant its formal description. The most striking difference, setting it apart from most species, is in the uropod and pleotelson morphology. While uropods that extend beyond the posterior margin of the pleotelson are not particularly rare in cymothoidans, in most species the endopod is distinctly wider than the exopod and, therefore, does not have a lanceolate shape as in †*Dysopodus gezei* gen. et sp. nov. (figure 7*a* versus figure 7*c*). There are, however, two occurrences of fossils from the Cretaceous of Mexico with a very similar uropod morphology.

The species †*Pseudoplakolana chiapaneca* (Bruce, Serrano-Sánchez, Carbot-Chanona and Vega, 2021) comb. nov. from the Lower Cretaceous Sierra Madre Formation in the Mexican state of Chiapas has similarly shaped uropods, endopods and exopods that extend even further beyond the posterior margin of the pleotelson. Apart from the even more elongated uropods, †*P. chiapaneca* comb. nov. differs from †*Dysopodus gezei* gen. et sp. nov. in having a narrower pleotelson with a distinctly pointed posterior tip on the sides of which there are slightly concave portions of the pleotelson margin (figure 7*b* [51]). Originally described as *Plakolana chiapaneca* Bruce, Serrano-Sánchez, Carbot-Chanona and Vega, 2021, the species was thought to be the oldest representative of †*Plakolana* Bruce, 1993. *Plakolana* comprises six extant species from Australia, Tasmania and Papua New Guinea [51], which are morphologically well recognizable by their enlarged, posteriorly produced lateral aspects of pleonite 3 bearing a distinct posterolateral incision ([67] fig. 7a), which has already been proposed as a putative apomorphy of *Plakolana* [68]. While fossils of †*P. chiapaneca* show posteriorly produced lateral aspects of pleonite 3, the distinct incision shared by the extant *Plakolana* species is neither described for the Mexican fossils [51] nor apparent from the figures, where the image resolution is insufficient to study such details. Further, the most conspicuous morphological feature of †*P. chiapaneca*—the elongated uropods with lanceolate endopods—is not present in the extant species of *Plakolana*. Therefore, the previously proposed affinity of †*P. chiapaneca* with *Plakolana* and its implications for the evolutionary history of *Plakolana*, such as a past worldwide distribution [51], are not justifiable in the absence of further morphological evidence.

Another fossil from the Lower Cretaceous of Mexico, reported from the Tlayua Formation in the state of Puebla ([69] fig. 9), shows a great similarity in its uropod morphology to both †*Dysopodus gezei* gen. et sp. nov. and †*Pseudoplakolana chiapaneca* comb. nov. Curiously, the fossil specimen has so far not been identified as an isopod, likely because the pleotelson appears to be not preserved. Despite the absence of a pleotelson for comparison, the uropods can be described as conspicuously elongated by comparison with other body parts. While a detailed study of the specimen is pending, the great similarity and the similar age and palaeo-geographical proximity already suggest a close affinity or conspecificity with †*Pseudoplakolana chiapaneca* comb. nov.

### 4.4. Comparison with extant species

There are also extant species with a similar uropod morphology, which coincidentally can all be found in freshwater. *Catailana whitteni* Messana, 2020, is known from a karstic freshwater-bearing cave in southern China, more than 300 km away from the coast [64]. While the uropod morphology is very similar to †*Dysopodus gezei* gen. et sp. nov., the body of *C. whitteni* is more elongate, the pleon tergites are curved to the ventral side and the pleotelson is truncated, with an almost straight posterior margin (figure 7*d*). *Typhlocirolana gurneyi* Racovitza, 1912, reported from freshwater in a cave in northern Algeria, also has very elongated uropod rami, of which the endopod has a slightly concave lateral margin, yet it differs from †*Dysopodus gezei* gen. et sp. nov. in having a more narrow pleotelson with a distinctly angled posterior tip (figure 7*e*). *Turcolana lepturoides* Prevorčnik, Konec and Sket, 2016, discovered in a freshwater stream in a cave on the Greek mainland, likely has the proportionally longest and narrowest (styliform) uropod rami in all non-parasitic cymothoidans and, by this, also distinctly differs from †*Dysopodus gezei* gen. et sp. nov., as well as by its semicircular pleotelson posterior margin (figure 7*f*; [66]).

### 4.5. Implications for the origin of related freshwater species

Cymothoida consists of mostly marine species. Nevertheless, there are numerous species that occur in freshwater. Of these, many are either parasites of fish (Cymothoidae) or crustaceans (Epicaridea) [4]. Of the remaining species (‘Cirolanidae’), the overwhelming majority are hypogean, meaning that they live in water-filled caves and groundwater [9], whereas there are only a few freshwater species recorded from rivers and lakes. The scarcity of extant ‘cirolanid’ freshwater species highlights the importance of the discovery of †*Dysopodus gezei* gen. et sp. nov., as an extinct species with robust support for a true freshwater palaeohabitat, not only for the evolutionary history of above-ground but also below-ground freshwater species.

In general, most freshwater isopod ingroups have a rather narrow geographical range, with few exceptions, such as the group Asellidae, which houses a large number of species in Eurasia and North America [4]. This is in accordance with the limited potential to spread beyond watersheds, which must be assumed for lineages adapted to a freshwater environment. While there is evidence suggesting that some extant groups like Phreatoicidea have colonized freshwater as early as in the Palaeozoic [70], the absence of extant freshwater species does not allow the conclusion that all freshwater species share a common freshwater ancestor, especially when the extant species are separated by ecological barriers such as watersheds or seawater. This is because their distribution can also be explained by independent freshwater colonizations, followed by a subsequent extinction of related marine species [4,70]. Such considerations naturally also apply to the colonization of cave and groundwater habitats.

While there are no apparent macroscopic morphological adaptations of cymothoidans to life in freshwater, and the epigean freshwater species share a similar appearance to their marine counterparts [e.g. [71]], hypogean species do differ from their above-ground (epigean) relatives. These differences can be characterized as a combination of rudimentation and the emergence of novel features [72]. The most obvious difference is the extreme rudimentation or complete lack of eyes and the lack of pigmentation in the underground species [72]. The hypogean species are also in general more slender, often have a smooth cuticula and have longer appendages. Their slenderness and the presence of various sensory structures can be interpreted as a compensation for their lack of visual perception [72]. The hypogean species are often found in karstic landscapes, not too far from the coastline [9]. Occurrences of hypogean cymothoidans span a large geographical area, including not only large parts of the peri-Mediterranean [66,73–78], East Africa [79,80], eastern North America [13,81], the Caribbean [82,83] and Mexico [84] but also China [64] and Australia ([85; figure 1*d*).

The published literature agrees that the colonization of underground freshwater environments happened at least a few times independently within Cymothoida. This is corroborated by the occurrence of hypogean species in cymothoidan ingroups that include epigean species as well (e.g. *Anopsilana*) [72,76]. However, there is still no reliable estimate for the number of independent cave colonizations that would explain the diversity of currently known underground species. A rather extreme proposal originates from a phylogenetic analysis of morphological characters, stating that most currently known hypogean species, including species on either side of the Atlantic, originated from a single common ancestor that already showed distinct morphological adaptations towards an underground lifestyle [7,76]. However, this opinion is problematic from different perspectives. First, the phylogenetic analysis was performed ‘by hand’ (‘groundpattern’ method [86]; for criticism, see [87]). Second, it was based on characters prone to convergence, e.g. the rudimentation of eyes or the elongatedness of appendage elements, whose derived states can also be found in other isopod lineages [72]. Finally, there is the lack of a good model for the distribution of an ancestral cave population over such a large area, beyond obstacles like watersheds or karst-free areas, despite being partially explained by plate tectonics. The few phylogenetic studies on the relationship between hypogean species based on sequence data [88,89] also somewhat in conflict with the results of the ‘groundpattern’ analysis. Nevertheless, they share an important similarity. The trees of both the morphological ‘groundpattern’ analysis and the molecular phylogenetic studies have in common that they suggest close relationships between geographically very distant species (e.g. a close relationship between species of *Speocirolana* from North and Central America and species of *Sphaeromides* from Europe) [88,89]. In this regard, it needs to be noted that the molecular studies mostly included hypogean species and, therefore, are not informative about the relations of the therein-studied species to their epigean marine relatives, to whom they could potentially be closer related than to any of the underground species included in the phylogenetic studies.

Wägele’s [7,76] phylogenetic trees suggest that there have been only a few independent epi- to hypogean transitions within Cymothoida. In stark contrast, the ‘regression model’ [90] explains the origin of hypogean freshwater species by a process of ‘stranding’, where marine species first transition to a hypogeic lifestyle, then some of them adapt to decreasing salinity and, while staying in place, ‘strand’ when the coastline regresses [12,91]. This regression model allows us to link the occurrences of hypogean freshwater species to geological regression events and could thus be used to address evolutionary questions, as the phylogenetic relationships should reflect the geological events [12,92]. Vice versa, through the regression model, the occurrence of hypogean freshwater arthropods has also been suggested as a palaeo-coastline indicator, supposedly informative about whether or not, but also when an area was covered by seawater [12,93,94]. While the absolute aspect relies on the appropriateness of the regression model, the temporal aspect relies on phylogenetic information. The morphological evolution of hypogean freshwater cymothoidans has predominantly been interpreted under the assumption that the epigean marine relatives have acquired more derived character states than their underground counterparts due to the supposedly more stable hypogean environment [12] (but see [72] on the ecological stability of cave environments). Given the unclear polarity of many morphological characters within Cymothoida (e.g. [22]), it is not surprising that several relationships inferred in this manner have not been confirmed by molecular phylogenetic studies, suggesting a high degree of convergence [88,89]. Also, this does not take into account any above- or below-ground dispersal within fresh water because the core of the model is that the species stay in place after being disconnected from the marine environment [92]. While this model regards physical barriers like watersheds, it focuses on epigean–marine to hypogean-freshwater transitions where the epigean to hypogean/interstitial transition happens prior to the adaptation to a freshwater medium. The focus on this specific order of transitions, apart from the good fit between the geographic occurrence of freshwater cave species and the reconstructed movement of the coastlines through geological time, is rooted in the scarcity of epigean freshwater species in the respective area [12]. While for a considerable proportion of the hypogean freshwater ‘cirolanids’ the regression model offers a good explanation for their geographical occurrence, the existence of closely related species or conspecifics living in epigean freshwater weakens the regression model as a parsimonious explanation [92]. Likewise, the occurrence of cave or groundwater species outside of areas covered by the sea, at a time when the marine-to-freshwater transition is expected to have happened, can weaken the explanatory strength of the regression model [92]. This is, for example, the case for the North American cave-dwelling species *Antrolana lira*, which occurs in an area not covered by the sea since the Palaeozoic. Its distribution can either be explained by a dispersal through epigean rivers and streams [13] or by dispersal through hypogean water bodies inside a karstic environment [95].

As laid out above, while the regression model seems to fit well for many cave and ground-water species, especially in the peri-Mediterranean region, one of the core assumptions of the model is the absence of closely related epigean freshwater species, with ancestors that could have given rise to an underground population. This is where the discovery of †*Dysopodus gezei* gen. et sp. nov. has the potential to incite some controversy. The herein presented fossils are from a time (Early Cretaceous: Barremian) before the major marine regressions took place (Late Cretaceous) that, according to the regression models, isolated the hypogean isopods from the marine environment [11]. In addition, there are various environmental indicators from the fossil site that convincingly point towards a true freshwater lake palaeoenvironment (see §1.1). Representatives of †*D. gezei* gen. et sp. nov. lived on the continental northwestern portion of the closing Neo-Tethys (later to be the easternmost side of peri-Mediterranean; figure 1*e*). This makes the fossils stand out, as there are no records of cymothoidans from lakes or rivers for the entire peri-Mediterranean (electronic supplementary material, files S1 and 2; [96]).

†*Dysopodus gezei* gen. et sp. nov. does not show any morphological features that could be linked to subterranean lifestyle. On the contrary, the eyes appear well developed in the holotype, which is a good indicator of an epigean lifestyle, as the rudimentation of eyes appears to happen very fast over an evolutionary time frame [9]. However, the long and slender uropods resemble those of some underground species (figure 7*a* versus figure 7*d–f*), and it has been shown that many hypogean cymothoidans have developed longer and more slender appendages than their above-ground relatives [9], which could be an advantageous trait for an ancestor of subterranean species.

**Figure 8.**
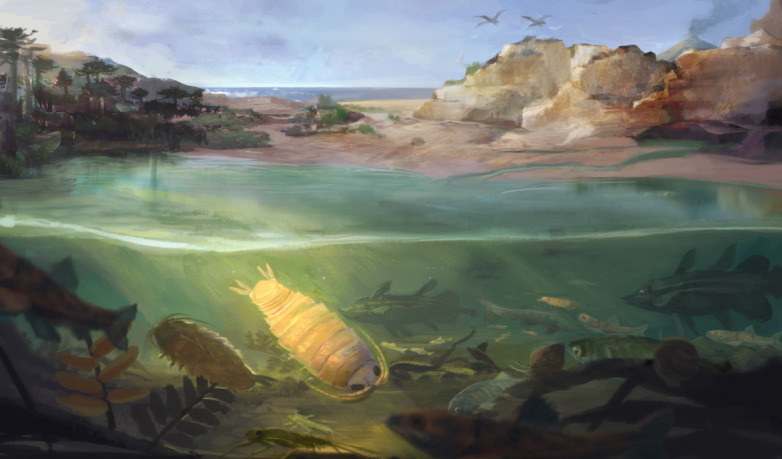
Palaeoenvironmental habitat reconstruction for †*Dysopodus gezei* gen. et sp. nov. (foreground) - a Barremian freshwater lake in the region of present-day Bkassine (Lebanon). Artwork by Aldrich Hezekiah.

The age, the palaeo-geographic location and the reconstructed freshwater lake habitat (illustrated in figure 8) combined have the potential to incite some controversy regarding the origin of some of the hypogean species found in the southern and eastern Mediterranean region. This is because, despite its currently uncertain phylogenetic position, †*D. gezei* gen. et sp. nov., or a closely related extant species, could have invaded subterranean habitats and given rise to one or more extant species that nowadays live in freshwater caves or groundwater. This is, of course, highly speculative, and there is no reason currently to favour this scenario over one where the epi- to hypogean transition happened before the adaptation to low salinity levels. Nevertheless, it should be considered that it might not have been necessary to have a widespread epigean freshwater fauna that persisted over long periods of time in order to give rise to one or more hypogean freshwater species. Furthermore, it should also be taken into account that Cymothoida has produced numerous independent freshwater lineages, among which many are found in its ingroups with parasitic forms, such as the fish parasites of Cymothoidae, which occur in rivers in Africa and South America [4,96] and are even present in the European fossil record [62]. This suggests that for cymothoidans, the evolutionary adjustment to low salinity levels might not represent a major obstacle and could have happened rather frequently. Also, the level of competition with other arthropod groups, such as insects, where many lineages nowadays have highly specialized aquatic larvae and highly mobile winged adults, might have been lower in the Mesozoic, allowing for more cymothoidan species to survive in epigean freshwater bodies. This could explain, albeit in a very speculative manner, the number of fossil cymothoidan remains associated with fresh- or brackish-water palaeohabitats (figure 1*d,e*; [16–18,20]).

## 5. Conclusion

The two herein presented fossils are interpreted to belong to a single species—†*Dysopodus gezei* gen. et sp. nov.—within Cymothoida, within which its exact position is unknown. However, it is evident that it does not belong to any of the parasitic or micro-predatory lineages of Cymothoida and strongly resembles extant species of ‘Cirolanidae’ (monophyly questionable, especially concerning its fossil record). †*Dysopodus gezei* gen. et sp. nov. is very reminiscent of another extinct species, †*Pseudoplakolana chiapaneca* gen. nov. et comb. nov., from the Lower Cretaceous of Mexico, as well as a not formally described fossil of a similar age also found in Mexico (likely closely related or conspecific with †*P. chiapaneca* gen. nov. et comb. nov.). Both Mexican fossils also feature slender uropods that protrude well beyond the posterior margin of the pleotelson, which, with the exception of †*D. gezei* gen. et sp. nov., are only found in a few non-parasitic species of Cymothoida, notably in several subterranean freshwater species.

The discoveries of the herein-described fossils represent a rare find of fossil isopods from a freshwater habitat. This puts the origin of extant non-parasitic freshwater cymothoidans into a new perspective. While this find does not disprove the colonization of cave and groundwater habitats through a disconnection of subterranean species by a regressing coastline, the presence of freshwater cymothoidans in the eastern Tethyan region during the Early Cretaceous shines a different light on the origin of the extant freshwater fauna. With the potential to preserve fine morphological details, additional specimens of this species could provide more morphological details that then could be used to draw more precise conclusions about the relationship of the Cretaceous freshwater species with the extant cave and groundwater fauna.

## Data Availability

The data has been submitted as electronic supplementary material [97].
